# Direct Evidence that Myocardial Insulin Resistance following Myocardial Ischemia Contributes to Post-Ischemic Heart Failure

**DOI:** 10.1038/srep17927

**Published:** 2015-12-14

**Authors:** Feng Fu, Kun Zhao, Jia Li, Jie Xu, Yuan Zhang, Chengfeng Liu, Weidong Yang, Chao Gao, Jun Li, Haifeng Zhang, Yan Li, Qin Cui, Haichang Wang, Ling Tao, Jing Wang, Michael J Quon, Feng Gao

**Affiliations:** 1 Department of Aerospace Medicine, The Fourth Military Medical University, Xi’an 710032, China; 2 Department of Physiology, The Fourth Military Medical University, Xi’an 710032, China; 3 Department of Cardiac Surgery, Xijing Hospital, The Fourth Military Medical University, Xi’an 710032, China; 4 Department of Nuclear Medicine, Xijing Hospital, The Fourth Military Medical University, Xi’an 710032, China; 5 Department of Cardiology, Xijing Hospital, The Fourth Military Medical University, Xi’an 710032, China; 6 Division of Endocrinology, Diabetes and Nutrition, University of Maryland School of Medicine, Baltimore, MD 21201, USA

## Abstract

A close link between heart failure (HF) and systemic insulin resistance has been well documented, whereas myocardial insulin resistance and its association with HF are inadequately investigated. This study aims to determine the role of myocardial insulin resistance in ischemic HF and its underlying mechanisms. Male Sprague-Dawley rats subjected to myocardial infarction (MI) developed progressive left ventricular dilation with dysfunction and HF at 4 wk post-MI. Of note, myocardial insulin sensitivity was decreased as early as 1 wk after MI, which was accompanied by increased production of myocardial TNF-α. Overexpression of TNF-α in heart mimicked impaired insulin signaling and cardiac dysfunction leading to HF observed after MI. Treatment of rats with a specific TNF-α inhibitor improved myocardial insulin signaling post-MI. Insulin treatment given immediately following MI suppressed myocardial TNF-α production and improved cardiac insulin sensitivity and opposed cardiac dysfunction/remodeling. Moreover, tamoxifen-induced cardiomyocyte-specific insulin receptor knockout mice exhibited aggravated post-ischemic ventricular remodeling and dysfunction compared with controls. In conclusion, MI induces myocardial insulin resistance (without systemic insulin resistance) mediated partly by ischemia-induced myocardial TNF-α overproduction and promotes the development of HF. Our findings underscore the direct and essential role of myocardial insulin signaling in protection against post-ischemic HF.

Heart failure (HF) remains a leading cause of morbidity and mortality in the developed world. Laboratory and clinical studies document strong associations between insulin resistance and HF in both animal models and humans. In the Framingham Heart Study, diabetes is an independent risk factor for HF^1^. Diabetes, obesity, and associated insulin resistance triples risk of cardiovascular complications^2^,^3^. In non-diabetic patients with HF, insulin resistance is also a pathophysiological hallmark^4^,^5^. A 9-year prospective study of ~12,000 Swedish adults without prior HF demonstrates that systemic insulin resistance *per se* (without diabetes) predicts HF^6^. In the Cardiovascular Health Study, systemic insulin resistance is found to be associated with abnormal cardiac structure and risk of HF^7^. However, in these studies, only systemic insulin resistance is evaluated without considering myocardial insulin resistance. Given the importance of myocardial insulin signaling in protection against ischemia-induced injury and subsequent cardiac dysfunction/remodelling^8^, local myocardial insulin resistance *per se* and its association with post-ischemic HF are inadequately investigated. Therefore, rigorous investigation of ischemic HF in the context of impaired myocardial insulin signaling/action is warranted.

Tumor necrosis factor-α (TNF-α) is a pro-inflammatory cytokine that promotes ischemic myocardial injury and cardiac dysfunction^9^. After myocardial infarction (MI), TNF-α is locally released from ischemic cardiomyocytes and remains markedly elevated in advanced HF^10^. TNF-α impairs insulin signaling and action, in part, by increasing serine phosphorylation of insulin receptor substrate-1 (IRS-1). This impairs insulin-stimulated tyrosine phosphorylation of IRS-1 that reduces binding of phosphatidylinositol 3-kinase (PI3K) to IRS-1. Consequently, activation of PI3K and downstream signaling molecules essential for regulation of glucose metabolism is impaired^11^. In peripheral insulin targets including skeletal muscle and liver (*in vitro* and *in vivo*), TNF-α promotes insulin resistance by similar mechanisms^12^,^13^. Thus, elevated local and/or circulating levels of TNF-α may contribute directly to myocardial insulin resistance.

Our previous studies have demonstrated that insulin exerts cardioprotective effects via PI3K/Akt-dependent survival signaling pathways that promote metabolic, anti-apoptotic and anti-inflammatory actions in various animals, including canine model of myocardial ischemia/reperfusion^14^,^15^,^16^. Insulin-triggered survival signaling and actions in the heart are blunted in both systemic and myocardial insulin resistance^17^. Systemic insulin resistance (that likely includes myocardial insulin resistance) increases infarct size in ischemic hearts leading to reduced functional recovery.

We therefore hypothesize that myocardial insulin resistance *per se* contributes to progression of post-ischemic HF. To test our hypothesis, we developed a rodent model of MI to study the effects of local cardiac TNF-α overexpression (adenoviral) and blockade (etanercept), insulin treatment, and cardiac insulin receptor signaling with respect to post-MI heart structure/function and subsequent HF.

## Results

### Cardiac dysfunction and remodeling after MI

**Figure 1:**
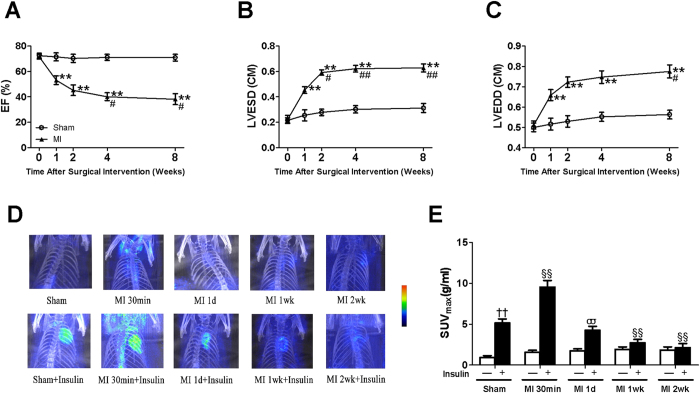
Cardiac function and dimensions, and myocardial insulin sensitivity in rats subjected to sham or myocardial infarction. (**A**) Ejection fraction (EF) was progressively reduced in rats following MI over 8 wk when compared with sham rats. (**B**) Left ventricular end-systolic dimensions (LVESD) and (**C**) Left ventricular end-diastolic dimensions (LVEDD) were progressively elevated in rats with MI over 8 wk compared with sham. (**D**) Representative microPET/CT images of rats with sham or MI over 2 wk without or with insulin stimulation. (**E**) Quantification of maximum standardized glucose uptake values (SUV_max_) obtained from multiple independent experiments as depicted in panel D. Data are mean ± SEM of 8 independent experiments. “Sham” means “non-MI + saline” at 30 min post-operation. **P < 0.01 vs. sham, ^#^*P* < 0.05, ^##^*P* < 0.01 vs. MI 1 wk, ^††^*P* < 0.01 vs. vehicle in sham, ^§§^*P* < 0.01 vs. insulin in sham, ^σσ^*P* < 0.01 vs. vehicle in MI 1d.

We performed serial echocardiography in rats before MI, and 1, 2, 4, and 8 wk post-MI. Progressive LV dilation and heart dysfunction were observed over time (Fig. 1A–C). When compared with sham-operated rats, substantial reduction in EF (53 ± 3%) and increased LV diameter (LVESD 0.45 ± 0.03 cm) were observed 1 wk post-MI. These abnormalities worsened over time with maximal LV dysfunction (EF 40 ± 3%) and dilation (LVESD 0.62 ± 0.03 cm) achieved and maintained 4 to 8 wk post-MI.

### Myocardial insulin resistance occurred early in ischemic HF

Basal and insulin-stimulated FDG uptake in rat hearts was assessed 30 min, 1 d, 1 wk, and 2 wk after MI (Fig. 1D). At baseline, FDG uptake in hearts of rats assigned to MI or sham groups was comparable. Insulin injection (10 U/kg) produced robust increases in cardiac FDG uptake in sham rats 30 min after surgery (Fig. 1E, left bars). By contrast, insulin-stimulated myocardial FDG uptake was even higher at the 30 min time point in rats post-MI (Fig. 1E). However, after 30 min, over time, progressive decrease in insulin-stimulated myocardial FDG uptake consistent with progressive increase in myocardial insulin resistance was observed in hearts of rats 1 d, 1wk, and 2 wk post-MI (Fig. 1D,E).

Typically, myocardial insulin resistance follows, in parallel, changes in systemic whole body insulin resistance^18^. In our model system with MI, we did not observe any significant differences in fasting plasma glucose (FPG), fasting plasma insulin (FIN), quantitative insulin sensitivity check index (QUICKI), oral glucose tolerance test (OGTT), or insulin tolerance test (ITT) when sham-operated and post-MI rats were evaluated and compared at 1 or 2 wk post-MI (Supplement Figure S1). Thus, MI caused local myocardial insulin resistance without systemic insulin resistance or glucose intolerance.

### Characterization of myocardial insulin resistance after surgically-induced MI

**Figure 2:**
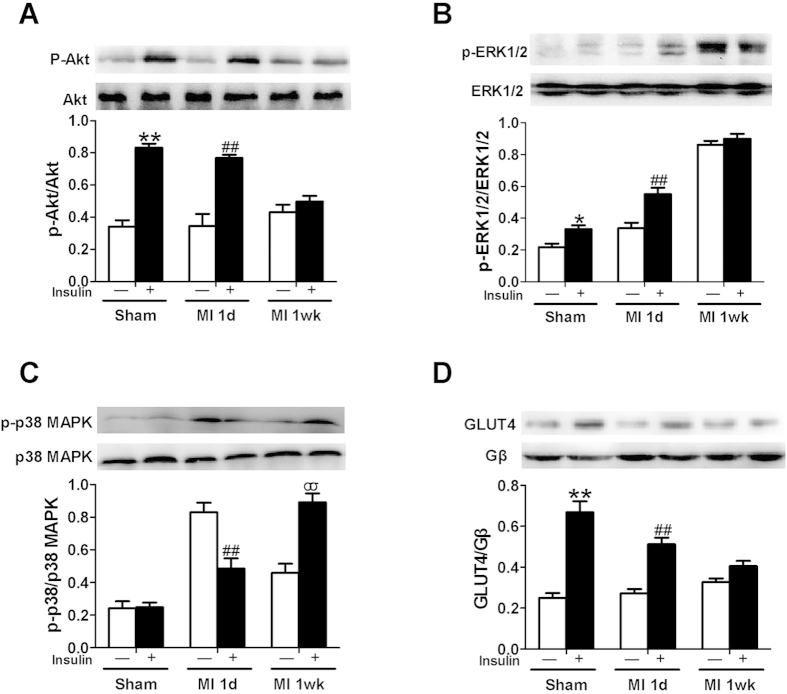
Insulin signaling and cardiac plasma membrane GLUT4 in sham or MI rats. (**A**) Insulin stimulated phosphorylation of Akt was almost abolished 1 wk after MI. (**B**) Both basal and insulin stimulated phosphorylation of ERK1/2 was enhanced 1 wk after MI. In addition, 1 wk after MI, insulin did not further stimulate the elevated basal phosphorylation of ERK1/2. (**C**) Insulin treatment significantly increased phosphorylation of p38 MAPK 1 wk after MI. (**D**) Insulin-stimulated increase in cardiac plasma membrane GLUT4 was impaired 1d after MI and abolished 1 wk after MI compared with sham. The bar graphs below the respective immunoblots represent quantification of multiple independent experiments from 3 animals (mean ± SEM). “Sham” means “non-MI + saline” at 30 min post-operation. *P < 0.05, **P < 0.01 vs. vehicle in sham, ^##^*P < *0.01 vs. vehicle in MI 1d, ^σσ^*P* < 0.01 vs. insulin in sham.

Elevated basal Akt phosphorylation (without insulin stimulation) was induced by myocardial ischemia. This is well demonstrated both in our laboratory and others^19^,^20^. Insulin stimulation of either sham or post-MI rats 1 d after operation caused robust increases in cardiac Akt phosphorylation. However, 1 wk post-MI, insulin-stimulated Akt phosphorylation was barely detectable (Fig. 2A). With respect to ERK 1/2 phosphorylation, both basal and insulin stimulated phosphorylation were a little higher in post-MI rats at 1 d when compared with sham animals. By 1 wk post-MI, basal ERK 1/2 phosphorylation was extremely elevated with no detectable insulin-stimulated increase (Fig. 2B). For p38 MAPK phosphorylation, there was no detectable insulin-stimulated increase in hearts of sham animals at baseline. Basal p38 MAPK phosphorylation was greatly increased in hearts 1 d post-MI, while insulin stimulation reduced phosphorylation. At 1 wk post-MI, basal phosphorylation levels were intermediate between those in hearts from sham rats and 1 d post-MI rats, while insulin now stimulated a robust increase in p38 MAPK phosphorylation (Fig. 2C). We next evaluated cell surface GLUT4 in heart as a downstream metabolic action (Fig. 2D). In sham animals, as expected, insulin stimulation caused increased GLUT4 translocation to the myocardial cell surface that predicts insulin-stimulated glucose uptake (Fig. 2D). In post-MI animals, insulin also stimulated GLUT4 translocation. However, the magnitude of this effect was smaller 1 d post-MI, and almost absent 1 wk post-MI. Thus, in hearts of rats subjected to MI, we observed local myocardial insulin resistance at the signaling level with respect to insulin-stimulated Akt phosphorylation, and at the functional level with respect to GLUT4 translocation (and presumably glucose uptake and metabolism).

### Overexpression of TNF-α in heart mimics myocardial insulin resistance and HF caused by surgical MI

**Figure 3:**
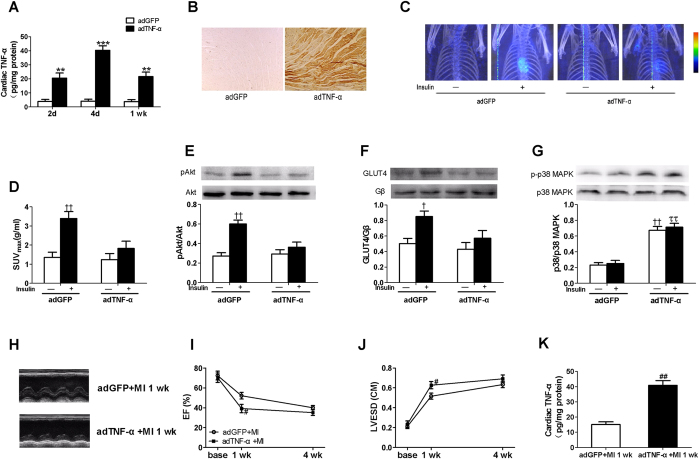
Local adenoviral expression of TNF-α in the heart resulted in myocardial insulin resistance in non-MI hearts and led to contractile impairment of the heart. (**A**) Tumor necrosis factor (TNF)-α concentrations in myocardium 2, 4, and 7 d after infection. (**B**) Myocardial TNF-α was demonstrated by immunohistochemistry staining in the left ventricular 7 days post-adenovirus infection. Adenoviral expression of TNF-α in the heart abolished insulin-stimulated myocardial glucose uptake **(C**,**D)**. Insulin-stimulated increase in Akt (**E)** and cardiac plasma membrane GLUT4 (**F**) was impaired in adTNF-α-treated rats. (**G**) P38 MAPK phosphorylation was increased in hearts infected with TNF-α with or without insulin stimulation. (**H**) Representative echocardiography images (major finding is I and J). Reduced Ejection fraction (**I**, EF) and elevated Left ventricular end-systolic dimensions (**J**, LVESD) were observed in the TNF-α overexpressed hearts at 1 wk after MI when compared with adGFP-treated rats. (**K**) Cardiac TNF-α levels in adTNF-α-treated or adGFP-treated rats at 1 wk after MI. Data are mean ± SEM of 8 independent experiments. **P < 0.01 vs. adGFP, ^†^*P* < 0.05, ^††^*P* < 0.01 vs. vehicle in adGFP, ^ττ^*P* < 0.01 vs. insulin in adGFP, ^#^*P* < 0.05, ^##^*P* < 0.01 vs. adGFP + MI.

Chronic inflammation mediated by pro-inflammatory cytokines including TNF-α may contribute to systemic insulin resistance^21^. However, effects of local cardiac overexpression of TNF-α on myocardial insulin resistance is unknown. Local overexpression of TNF-α in heart significantly increased cardiac TNF-α levels with the highest levels achieved 4 d after adenovirus infection (Fig. 3A). By 1 wk post-adenovirus infection, TNF-α levels in hearts infected with adTNF-α were declining back to levels seen 2 d post-adenovirus infection. Importantly, no detectable changes in circulating serum TNF-α were observed at 2 d and 4 d after adenovirus infection (lower limit of detection for the assay used = 25 pg/mL). This confirms that TNF-α overexpression was strictly limited to the heart reported above. Myocardial TNF-α was determined by immunohistochemistry staining in the left ventricular 7 d post-adenovirus infection. Compared with the adGFP-treated animals, TNF-α immunostaining was obviously present in adTNF-α-treated rats (Fig. 3B). Insulin injection (10 U/kg, i.p.) stimulated increased FDG uptake in hearts of control adGFP-treated rats (Fig. 3C,D). This was substantially blunted in adTNF-α-treated rats 1 wk post-adenovirus infection. When compared with adGFP-treated control animals, insulin-stimulated myocardial Akt phosphorylation (Fig. 3E) and translocation of GLUT4 (Fig. 3F) were severely blunted in hearts from adTNF-α-treated animals 1 wk post-adenovirus infection. This was accompanied by increased p38 MAPK phosphorylation in adTNF-α-treated hearts (Fig. 3G). Echocardiographic examination at baseline revealed no significant differences in EF and LVESD when adTNF-α-treated and adGFP-treated rats were evaluated (EF 70 ± 5 vs. 73 ± 4%) at baseline (post-adenovirus infection without MI). However, substantial LV dysfunction (EF 39 ± 4 vs. 52 ± 3%) and dilation (LVESD 0.63 ± 0.04 vs. 0.52 ± 0.03 cm) were observed in hearts overexpressing TNF-α when compared with adGFP-treated animals 1 wk post-MI (Fig. 3I,J). TNF-α levels were still significantly higher in adTNF-α-treated rats at 1 wk after MI when compared with adGFP-treated animals (Fig. 3K). Taken together, these results demonstrate that local cardiac overexpression of TNF-α mimics myocardial insulin resistance, and exacerbates poor heart function observed with surgically-induced MI.

### TNF-α antagonism improved local myocardial insulin resistance caused by MI

**Figure 4:**
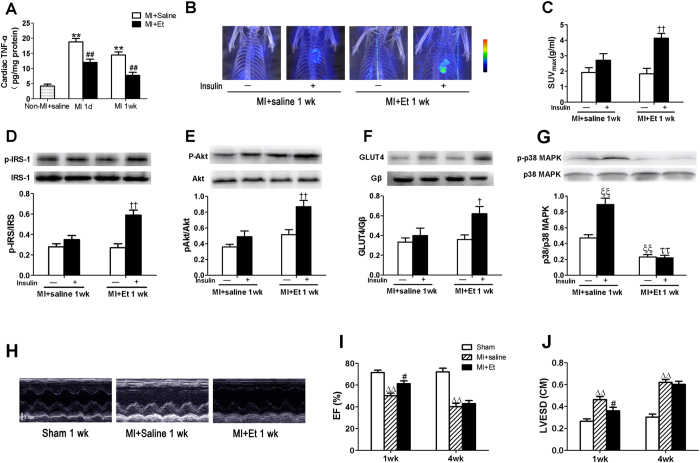
Systemic etanercept treatment reduced myocardial TNF-α levels and improved myocardial insulin sensitivity and action at 1 wk post-MI. (**A**) MI caused a significant increase in myocardial TNF-α expression that was somewhat blunted in etanercept treated rats. Etanercept treatment enhanced insulin stimulated glucose uptake 1 wk after MI when compared with vehicle treated rats **(B**,**C)**. Insulin-stimulated increase in p-IRS-1 (**D**) p-Akt (**E)** and cardiac plasma membrane GLUT4 (**F**) was restored in etanercept-treated rats at 1 wk after MI. (**G**) P38 MAPK phosphorylation was decreased in etanercept-treated rats at 1 wk after MI. (**H**) Representative echocardiography images (major finding is I, J). (**I**) Ejection fraction (EF). (**J**) Left ventricular end-systolic dimensions (LVESD). Data are mean ± SEM of 8 independent experiments. **P < 0.01 vs. Non-MI + saline (Sham), ^#^*P* < 0.05, ^##^*P* < 0.01 vs. MI + saline, ^†^*P* < 0.05, ^††^*P* < 0.01 vs. vehicle in MI + Et 1wk, ^ξξ^*P* < 0.01 vs. vehicle in MI + saline 1wk, ^ττ^*P* < 0.01 vs. insulin in MI + saline 1wk, ^ΔΔ^*P* < 0.01 vs. Sham.

We next treated our animal model with etanercept to block endogenous TNF-α signaling and action. Our preliminary experiments have shown that the presence of etanercept at this dose (300 μg/250 g body weight) had no significant effects on myocardial TNF-α production and cardiac function in non-MI (sham) rats. Pretreatment with etanercept during the first week after MI repressed local myocardial TNF-α production (circulating serum TNF-α levels were undetectable) (Fig. 4A). This substantially restored insulin-stimulated IRS-1 phosphorylation (Fig. 4D), Akt phosphorylation (Fig. 4E), and GLUT4 translocation (Fig. 4F) as well as myocardial FDG uptake (Fig. 4B,C) 1 wk post-MI. Moreover, etanercept treatment significantly suppressed p38 MAPK phosphorylation without or with insulin stimulation 1 wk post-MI (Fig. 4G). Thus, opposing TNF-α action preserved normal insulin signaling and action in myocardium post-MI. Etanercept treatment improved cardiac function and inhibited LV dilation at 1 wk after MI, while there were no significant differences in cardiac function or LV dilation when hearts from saline and etanercept-treated animals were evaluated 4 wk post-MI (Fig. 4H–J). Thus, our data suggest that TNF-α plays an important role in myocardial insulin resistance resulting from MI.

### Early insulin treatment suppressed cardiac TNF-α production and improved myocardial insulin sensitivity and cardiac function post-MI

**Figure 5:**
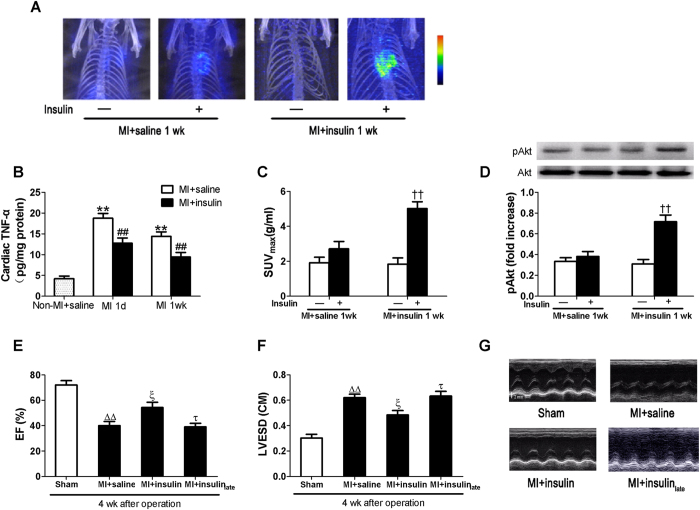
Early insulin treatment reduced cardiac TNF-α expression and improved myocardial insulin sensitivity and cardiac function following MI. (**B**) TNF-α expression in myocardium. Early insulin treatment significantly improved acute insulin-stimulated myocardial glucose uptake 1 wk after MI compared with vehicle treated rats **(A**,**C)**. Insulin-stimulated increase in Akt phosphorylation (**D)** was restored in early insulin-treated rats at 1 wk after MI. (**G**) Representative echocardiography images (major finding is E, F). Increased ejection fraction (**E**, EF) and reduced Left ventricular end-systolic dimensions (**F**, LVESD) were observed in early insulin-treated rats at 4 wk after MI when compared with saline-treated or late insulin-treated animals. Values presented are mean ± SEM (n = 8 per group). **P < 0.01 vs. Non-MI + saline, ^#^*P* < 0.05, ^##^*P* < 0.01 vs. MI + saline, ^††^*P* < 0.01 vs. insulin in MI + saline 1wk, ^ΔΔ^*P* < 0.01 vs. Sham, ^ξ^*P* < 0.01 vs. MI + saline, ^τ^*P* < 0.05 vs. MI + insulin.

In a healthy context with intact PI3K signaling (e.g., normal insulin sensitivity with euglycemia), insulin treatment opposes pro-inflammatory signaling/actions while promoting anti-inflammatory signaling/actions in vasculature and macrophages to ameliorate cardiovascular pathophysiology^22^,^23^. Thus, insulin treatment may be helpful post-MI (assuming intact PI3K signaling). Consistent with our previous studies^23^, early insulin treatment given immediately after MI suppressed TNF-α production when compared with saline treatment (Fig. 5B). Our preliminary experiments have shown that insulin treatment had no significant effects on myocardial TNF-α production in non-MI (sham) rats. There were no significant changes in blood pressure and heart rate after the injection of insulin in MI rats. Early insulin treatment also improved subsequent insulin-stimulated myocardial FDG uptake and Akt phosphorylation 1 wk post-MI (Fig. 5A,C,D), and alleviated cardiac dysfunction and dilation 4 wk post-MI as evidenced by increased EF (Fig. 5E) and decreased LVESD (Fig. 5F). However, late insulin treatment initiated at 1 wk after MI when myocardial insulin resistance has developed exerted no beneficial effects on cardiac function and remodeling at 4 wk after MI.

### Aggravated post-ischemic LV dysfunction and remodeling in tamoxifen-induced cardiomyocyte-specific insulin receptor knockout (TCIRKO) mice

**Figure 6:**
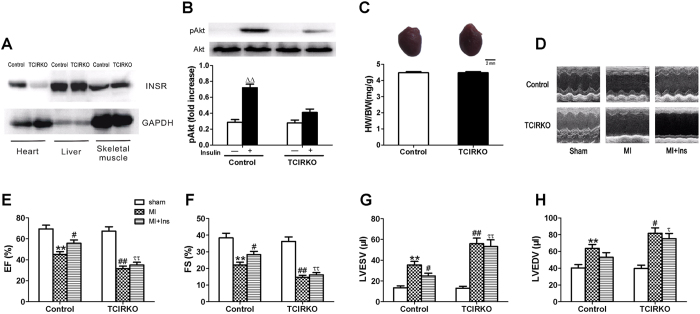
Aggravated LV dilation and dysfunction in tamoxifen-induced cardiomyocyte-specific insulin receptor knockout (TCIRKO) mice at 4 wk after MI. (**A**) Representative immunoblots demonstrating absence of insulin receptor (INSR) in cardiac muscle of TCIRKO mice. Insulin-stimulated myocardial Akt phosphorylation (**B**) was almost entirely blocked in TCIRKO mice. No detectable differences in heart weight/body weight (**C**, HW/BW) were observed between controls and TCIRKO. (**D**) Representative echocardiography images (major finding is E–H). TCIRKO mice developed decreased ejection fraction (**E**, EF) and fractional shortening (**F**, FS) and elevated left ventricular end-systolic volume (**G**, LVESV) and end-diastolic volume (**H**, LVEDV) compared with littermate controls at 4 wk after MI. Insulin administration improved EF and FS and reduced LVESV compared with controls at 4 wk after MI. These benefits were absent in TCIRKO mice. Data are mean ± SEM of 8 independent experiments. ^ΔΔ^*P* < 0.01 vs. vehicle in control, **P < 0.01 vs. sham in control, ^#^*P* < 0.05, ^##^*P* < 0.01 vs. MI in control, ^τ^*P* < 0.05, ^ττ^*P* < 0.01 vs. MI + Ins in control.

To substantiate the role of myocardial insulin resistance in progression of HF, we determined specific effects of cardiac insulin signaling independent of systemic insulin resistance. Tamoxifen injections (50 mg/kg/d) for 3 d in MHC-MerCreMer/IR^fl/fl^ mice induced Cre-mediated recombination to produce TCIRKO mice. Immunoblotting confirmed reduction of insulin receptor protein by ~78% in cardiac muscle (*P* < 0.01) but not in skeletal muscle or liver (Fig. 6A). Insulin-stimulated myocardial Akt phosphorylation was almost entirely blocked in TCIRKO mice (Fig. 6B). No significant differences in heart weight/body weight were observed when control and TCIRKO mice were compared (Fig. 6C). Overnight-fasted (16 h) mice were injected i.p. with glucose (2 g /kg) for glucose tolerance tests (GTT) or insulin (0.75 U/ kg) for insulin tolerance tests (ITT). No significant differences in GTT or ITT were observed among control and TCIRKO mice (Supplement Figure S2). Thus, systemic insulin sensitivity and glucose tolerance was not altered in TCIRKO mice. No significant differences in cardiac function or LV dimensions were observed when TCIRKO sham mice were compared with control mice (MHC-MerCreMer/IR^+/+^). By contrast with littermate controls, TCIRKO mice had exaggerated LV dysfunction and dilation 4 wk post-MI (EF 31.5 ± 2.4 vs. 45.3 ± 2.7%, LVESV 31.5 ± 2.4 vs. 45.2 ± 2.7 μl, both *P* < 0.05) (Fig. 6E–H). Insulin treatment improved cardiac function and inhibited LV dilation only in littermate controls 4 wk post-MI (*P* < 0.05). These benefits were absent in TCIRKO mice (Fig. 6E–G). Thus, myocardial insulin resistance due to conditional knockout of heart insulin receptors alone was sufficient to greatly exacerbate post-ischemic HF.

## Discussion

We have made three major findings in the present study. First, we observed that myocardial insulin resistance occurred as early as 1 wk following surgically-induced MI in rats, while systemic insulin sensitivity and glucose tolerance remained normal. Second, this myocardial insulin resistance was mediated partly by increased TNF-α production from the ischemic heart. Third, insulin treatment itself opposed myocardial insulin resistance caused by MI. This ameliorated post-ischemic cardiac dysfunction, and subsequent HF. Moreover, cardiac specific insulin receptor knockout in adult mice exacerbated cardiac dysfunction and the development of HF post-MI. Indeed, the effects of cardiac-specific insulin receptor deletion occurred without causing changes in systemic insulin sensitivity. This determines an essential role of myocardial insulin signaling in protection against ischemic HF.

A close link between HF and systemic insulin resistance in humans has been recognized for decades. Most large clinical trials on HF report incident rates of diabetes of 15–35%^24^. Systemic insulin resistance often precedes development of HF suggesting that altered metabolic environment results in myocardial dysfunction and HF. In 1997, Botker *et al.* were the first to report myocardial insulin resistance in patients with metabolic syndrome^25^. Both systemic and cardiac insulin resistance are observed in non-diabetic patients or animals with moderate to advanced HF in most contexts^26^,^27^. Cardiac insulin resistance may occur as a result of systemic insulin resistance^18^,^28^. Amorim *et al.* have found that myocardial insulin resistance occurred at 2 wk after MI^29^, when the rats have developed heart failure (ejection fraction < 50%). In the present study, we found that myocardial insulin resistance occurred as early as 1 wk after MI (reduced insulin-stimulated GLUT4 translocation and FDG uptake) before the occurrence of heart failure (HF) without any detectable change in systemic insulin sensitivity, when ejection fraction was still higher than 50%. Thus, myocardial insulin resistance alone may be an early event in the development of ischemic HF independent of systemic insulin resistance and glucose intolerance in certain contexts.

One of the key characteristics of molecular signaling mechanisms underlying metabolic insulin resistance in the clinical setting is selective impairment in PI3K/Akt signaling pathways while other major branches of insulin signaling including Ras/MAPK (ERK1/2, p38MAPK and JNK) pathways remain intact or are even enhanced^30^,^31^,^32^,^33^. In the presence of myocardial insulin resistance 1 wk after MI, we observed blunted insulin stimulation of Akt and ERK1/2 phosphorylation with concomitant enhanced p38 MAPK phosphorylation characteristic of pathway-selective insulin resistance. Local TNF-α overexpression caused cardiac insulin resistance while exacerbating functional and structural sequelae of post-MI. As TNF-α can induced self expression in some pathological conditions^34^,^35^, etanercept treatment may block TNF-α-induced self expression after MI and then repressed local myocardial TNF-α production. Moreover, etanercept treatment partially restored myocardial insulin sensitivity and improved cardiac function at 1 wk after MI, but did not improve cardiac function and remodeling at 4 wk after MI. The reason for this may be that beneficial cardiac effects with etanercept early after MI is offset by its adverse cardiovascular effects such as potentiating platelet–monocyte aggregation and causing TNF-α imbalance in the long-term development of ischemic heart failure^36^,^37^. It should be noted that the mechanisms of myocardial insulin resistance post-MI are multi-factorial and complex. TNF-α overproduction is only one of the important mechanisms and further study still needs to be done to reveal the complex mechanisms.

Our study showed that insulin treatment activated Akt and ERK1/2, while reducing phosphorylation of p38 MAPK 1 d after MI. Thus, insulin therapy at the appropriate time can reverse pathophysiology underlying pathway-selective insulin resistance in heart that contributes to ischemic damage, myocardial dysfunction and HF. It seems likely that early insulin therapy improves insulin signaling and action, in part, through rebalancing insulin signaling between PI3K/Akt and MAPK insulin signaling branches to mediate anti-inflammatory actions opposing detrimental TNF-α actions in heart. Insulin treatment also exerts anti-apoptotic and anti-oxidative/nitrative stress effects that complement insulin anti-inflammatory actions^16^. This is supported by recent IMMEDIATE trial that immediate GIK administration was associated with lower rates of the composite outcome of cardiac arrest or in-hospital mortality in patients with ST segment elevation^38^. Our study further showed that late insulin administration (1 wk after MI) failed to exert beneficial effects, which may be due to blunted insulin-stimulated Akt phosphorylation.

To further confirm the role of myocardial insulin resistance in the development of ischemic HF, we used TCIRKO mice to achieve temporal control of insulin receptor disruption in the heart. This is an important advantage over the previous CIRKO model where absence of cardiac insulin receptors during development has resulted in cardiac pathology as shown by decreased myocyte size and modestly reduced cardiac function even at baseline^39^. In this study, no significant differences in heart weight/body weight ratio and cardiac function were observed when control and TCIRKO mice were compared at baseline. Importantly, loss of cardiac insulin receptors led to aggravated LV dysfunction and LV dilation after MI (without altering systemic insulin sensitivity), suggesting that myocardial insulin resistance contributes to the progression of ischemic HF. Taken together, our data showed that myocardial insulin resistance is not only a result of MI but also a contributing factor of cardiac dysfunction after MI.

In the clinical setting, antidiabetic agents aim to improve systemic insulin sensitivity, while some of them exert no beneficial effects on HF patients^40^. Our findings reveal that myocardial insulin resistance occurs early in the development of HF, which is independent of systemic insulin resistance, and contributes to the development of HF. Therefore, the intervention specifically targeted against myocardial insulin resistance may represent a potential therapeutical strategy for HF.

In summary, myocardial insulin resistance, independent of systemic insulin resistance, is an early event in the development of ischemic HF following MI. Impaired myocardial insulin action is at least partly mediated by overproduction of TNF-α. Our data suggest that potential therapeutic strategies targeted at reversing post-ischemic myocardial insulin resistance specifically may prevent or delay the progression of HF. Our findings are potentially translatable to the prevention and treatment of ischemic heart disease and HF.

## Materials and Methods

### Myocardial infarction protocol

All animal experiments were performed in accordance with the National Institutes of Health Guidelines on the Use of Laboratory Animals and were approved by The Fourth Military Medical University Committee on Animal Research. Male Sprague-Dawley rats (200–230 g) were anesthetized with 3% pentobarbital sodium. As described^41^, myocardial infarction was initiated after exposing the heart through a left thoracotomy at the fourth rib by using 4–0 silk to ligate the left anterior descending coronary artery (LAD) permanently near its origin from the left coronary artery. In sham rats, the silk suture was passed underneath the left anterior descending artery without ligation. Then the sham rats received saline administration.

### Echocardiography measurements

Serial doppler echocardiography was performed with the ACUSON Sequoia 512 ultrasound machine (Siemens) before the operation and 1, 2, 4 and 8 wk after the operation. A 14-MHz probe was used to obtain two-dimensional, and M-mode imaging from parasternal short-axis view at the level of the papillary muscles and the apical four-chamber view. LV end-systolic and end-diastolic diameters (LVESD and LVEDD respectively) were measured. LV fractional shortening (FS) was calculated as (LVEDD–LVESD)/LVEDD × 100%. Left ventricular end-diastolic volume (EDV) and end systolic volume (ESV) were calculated according to the formula by Teichholz *et al.*^42^. Ejection fraction (EF) was calculated as (EDV–ESV)/EDV × 100%.

### Assessment of systemic insulin sensitivity

Fasting plasma glucose (FPG) was analyzed by using standard glucose oxidase methods in rats fasted for 12 h. Fasting plasma insulin (FIN) was measured by using an enzyme linked immunosorbent assay. Quantitative insulin sensitivity check index (QUICKI) was calculated for estimating systemic insulin sensitivity. QUICKI = 1/[log(FIN) + log(FPG)]^43^. Rats were also given an oral glucose (2 g/kg) challenge or an i.p. injection of insulin (0.5 U/kg) (Novo Nordisk, China) to assess whole body glucose tolerance and insulin sensitivity. Whole blood glucose levels were determined at 0, 30, 60, 90 and 120 min after glucose challenge for oral glucose tolerance test (OGTT), or at 0, 30, 60, 90, 120, and 240 min for insulin tolerance test (ITT) (tail clipping used to obtain blood samples).

### Assessment of insulin-stimulated myocardial FDG uptake

Insulin-stimulated myocardial [18F]-fluorodeoxyglucose (FDG) uptake was measured as reported^44^. Briefly, animals were intravenously injected with ~1 mCi of FDG (tail vein) and subsequent PET/CT images were acquired (microPET/CT scanner; Medison, Budapest, Hungary). Insulin stimulation was initiated in overnight fasted rats through i.p. injection of 10 U/kg insulin 30 min before beginning image acquisition. Insulin-stimulated myocardial FDG uptake was assessed using the standardized uptake value SUV = C/(D/M) where C is activity concentration in regions of interest (ROI), D is the injected dose, and M is the body weight. ROI of identical size were chosen on myocardium outside of the infarcted region. Data reported are the maximal value of SUV (SUVmax) during the last 30 min of scanning.

### Whole tissue extract and plasma membrane fractionation

Insulin stimulation was induced in fasted rats through i.p. injection of 10 U/kg insulin. After 30 min of insulin or saline injection, animals were euthanized and non-infarcted LV myocardial tissues were harvested. Whole tissue extract was prepared by homogenizing myocardium tissue in ice-cold lysis buffer. The lysates were centrifuged and the supernatants were retained. Heart plasma membrane (PM) fraction was performed as described previously^45^. Ventricle tissue was homogenized in buffer A containing (in mmol/L, pH 7.0): 10 NaHCO_3_, 5 NaN_3_, and then centrifuged at 7000 × g for 20 min. The pellet was resuspended in buffer B (10 mmol/L Tris-HCl, pH 7.4), and centrifuged at 200 × g for 20 min. The supernatant was gently layered on top of a 20% (vol/vol) percoll gradient in buffer C (in mmol/L: 255 sucrose, 10 Tris-HCl (pH 7.4), 2 EDTA) and centrifuged at 55,000 × g for 1 h. The band with density of 1.030 was aspirated and pelleted by centrifugation at 170,000 × g for 1 h and resuspended in buffer C as PM solution. Protein concentration of whole tissue or PM solution was determined using a BCA protein assay. Glucose transporter 4 (GLUT4) content in PM was determined by immunoblotting using standard methods.

### Immunoblotting

Whole tissue proteins or PM proteins were separated on a SDS-PAGE Electrophoresis System as described previously^46^. Primary antibodies used were: phospho-IRS-1 (Tyr1222), IRS-1, phospho-Akt (Ser 473), Akt, phospho-ERK1/2 (Thr202/Tyr204), ERK1/2, phospho-JNK (Thr183/Tyr185), JNK, GLUT-4 and Gβ from Cell Signaling Technology; phospho-p38 MAPK (Thr180/Tyr182) and p38 MAPK from Abcam.

### Overexpression of TNF-α by adenovirus infection

Serotype 5 adenoviral vectors encoding TNF-α were provided by GeneChem, Shanghai, China. In brief, the cDNA for TNF-α was cloned into pMD19-T simple vector and then transferred into pAdTrack-CMV, resulting in pAdTrack-TNF-α. The shuttle vectors were used to generate recombinant adenoviral vectors encoding TNF-α (Ad-TNF-α). Adenoviral vectors encoding green fluorescent protein (Ad-GFP) were used as control. Adenoviruses were purified by double cesium chloride gradient ultracentrifugation. Viral titer was determined by plaque assay and expressed as plaque-forming units (pfu).

Adenovirus-mediated gene transfer to cardiac myocytes was performed as previously described^47^. A 24-gauge catheter containing 0.1 ml of viral solution (7.5 × 10^9^ pfu) was advanced from the apex of the left ventricle to the aortic root. The aorta and pulmonary arteries were then clamped distal to the site of the catheter and the solution injected. The clamp was maintained for 45 sec while the heart is pumping against a closed system isovolumically. This allows the solution that contains the adenovirus to circulate down the coronary arteries and perfuse the heart without direct manipulation of the coronaries. After that, the clamp on the aorta and pulmonary artery was released. Insulin-stimulated myocardial FDG uptake and Akt phosphorylation and GLUT4 membrane translocation were measured as previously described in non-MI rats 1 wk after adenovirus infection.

### Determination of myocardial and serum TNF-α levels

Myocardial and serum TNF-α levels were measured as described previously^48^. Heart tissue samples for determination of myocardial TNF-α were obtained from the non-infarcted area of LV. The protein content of the samples was measured with the use of a protein assay kit (bovine serum albumin was used as a standard control). Quantitative expression of the TNF-α was assessed using a rat ELISA kit (Neobioscience Technology Co., Ltd). The sensitivity range of this assay is 25 pg/mL
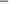
20000 pg/ml.

### Antagonism of TNF-α action with etanercept

Rats that had surgically induced MI (or control rats) were treated with the TNF-α inhibitor etanercept (Immunex, Seattle, Wash) by i.p. injection (300 μg/250 g body weight) 2 d prior to coronary artery ligation and every 2 d thereafter in the first week after MI. The dose and timing of etanercept administration was based on early studies^49^,^50^ and validated in our preliminary experiments. Insulin-stimulated myocardial FDG uptake and Akt phosphorylation and GLUT4 membrane translocation were measured as previously described for etanercept-treated rats at 1 wk after MI.

### Systemic insulin treatment

Subcutaneous daily injection with insulin (0.5 U/ml, 1ml/kg/d) was administrated in the first week after MI to investigate the effect of insulin on myocardial insulin sensitivity and the development of ischemic HF. Insulin-stimulated myocardial FDG uptake was measured as previously described in insulin-treated rats 1 wk after MI. Echocardiographic and hemodynamic analysis was performed in insulin-treated animals at 4 wk after MI.

### Inducible cardiomyocyte-specific insulin receptor knockout mice

We obtained mice from the Model Animal Research Centre of Nanjing University that were homozygous for loxP-flanked insulin receptor exon, and positive for tamoxifen-inducible Cre recombinase driven by the cardiomyocyte-specific α-myosin heavy chain (MHC) promoter (MHC-MerCreMer/IR^fl/fl^). Male mice aged 8–10 wk were injected intraperitoneally with tamoxifen (50 mg/kg) for 3 d to induce insulin receptor gene excision in cardiomyocytes. Age-matched male littermates (MHC-MerCreMer/IR^+/+^) were used as control animals. Littermate control mice were also treated with tamoxifen in an identical manner. After that, HW/BW ratio was measured and echocardiography was done before MI in knockout mice and controls.

### Myocardial infarction protocol in mice

Both knockout mice and controls were subjected to LAD ligation after the tamoxifen treatment. The mice were anesthetized with pentobarbital sodium (50 mg/kg i.p.) and connected to a rodent ventilator (volume-cycled ventilator supplying supplemental oxygen with a tidal volume of 2.5 ml and a respiratory rate of 120 beats/min) using a 20 G i.v. catheter. Via thoracotomy, the pericardial sac was opened and a 7–0 silk suture was passed beneath the root of LAD and tied to induce ischemia of the left ventricle. Finally the chest cavity and the skin were closed. All mice were maintained with free access to water and chow throughout the period of study. Echocardiography was performed again at 4 wk after MI in TCIRKO mice and controls.

### Statistical analyses

All values are presented as mean ± SEM. Differences among comparisons were evaluated with one-way ANOVA or two-way repeated measures ANOVA followed by Bonferroni post hoc tests where appropriate. Probabilities of p < 0.05 were considered statistically significant. Statistical tests were performed using GraphPad Prism software version 5.0 (GraphPad Software, Inc., San Diego, CA).

## Additional Information

**How to cite this article**: Fu, F. *et al.* Direct Evidence that Myocardial Insulin Resistance following Myocardial Ischemia Contributes to Post-Ischemic Heart Failure. *Sci. Rep.* **5**, 17927; doi: 10.1038/srep17927 (2015).

## Supplementary Material

Supplementary Information
