# Supplementary material for: Direct Evidence that Myocardial Insulin Resistance following Myocardial Ischemia Contributes to Post-Ischemic Heart Failure
Source: Sci Rep. 2015 Dec 14;5:17927. doi: 10.1038/srep17927 (PMC4677294; doi:10.1038/srep17927)
Supplement: Supplementary Information [file srep17927-s1.pdf]

Direct Evidence that Myocardial Insulin Resistance following Myocardial Ischemia Contributes to Post-Ischemic Heart Failure

Feng Fu [1,2†], Kun Zhao [3†], Jia Li [1], Jie Xu [1], Yuan Zhang [1], Chengfeng Liu[1], Weidong Yang [4], Chao Gao [5], Jun Li [1], Haifeng Zhang [1], Yan Li [5], Qin Cui [3], Haichang Wang [5], Ling Tao[5], Jing Wang [4], Michael J Quon[6]*, Feng Gao [1,5]*

([1]Department of Aerospace Medicine, [2]Department of Physiology, [3]Department of Cardiac Surgery, Xijing Hospital, [4]Department of Nuclear Medicine, Xijing Hospital, [5]Department of Cardiology, Xijing Hospital, The Fourth Military Medical University, Xi'an 710032, China; [6]Division of Endocrinology, Diabetes and Nutrition, University of Maryland School of Medicine, Baltimore, MD 21201, USA)

[†]These authors contributed equally to this study.

Supplemental Figures and Figure Legends

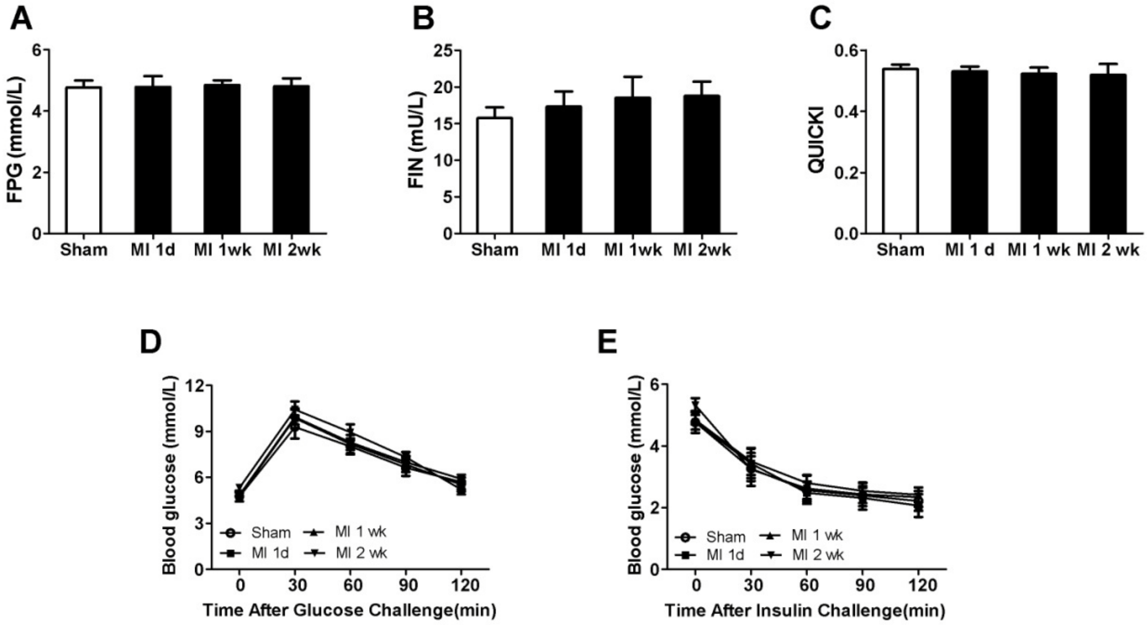

Supplement Figure S1. There were no significant changes in systemic insulin sensitivity between sham and MI rats. Systemic insulin sensitivity was evaluated by **A**, Fasting plasma glucose (FPG), **B**, Fasting plasma insulin (FIN), **C**, Quantitative insulin sensitivity check index (QUICKI), **D**, glucose tolerance test (GTT), and **E**, insulin tolerance test (ITT). Data are mean ± SEM of 8 independent experiments.

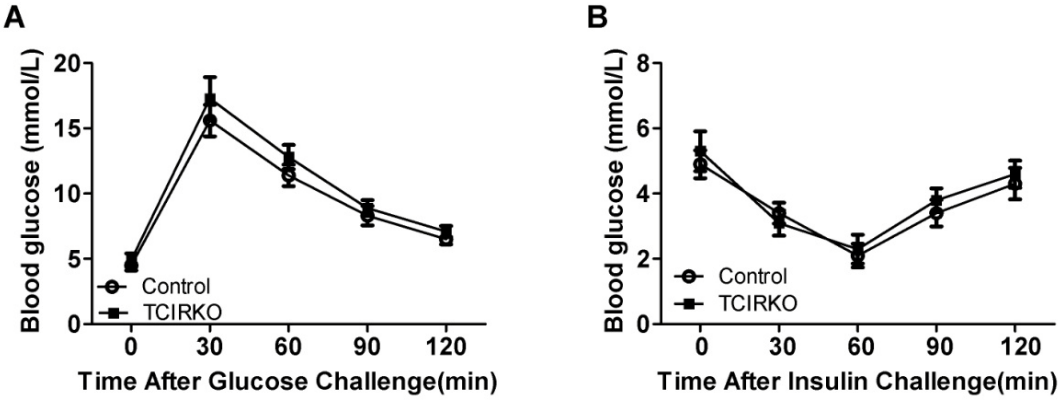

Supplement Figure S2. There were no significant differences in systemic insulin sensitivity between littermate controls and tamoxifen-induced cardiomyocyte-specific insulin receptor knockout (TCIRKO) mice. Systemic insulin sensitivity was evaluated by **A**, glucose tolerance test (GTT) and **B**, insulin tolerance test (ITT). Data are mean ± SEM of 8 independent experiments.

Figure 3

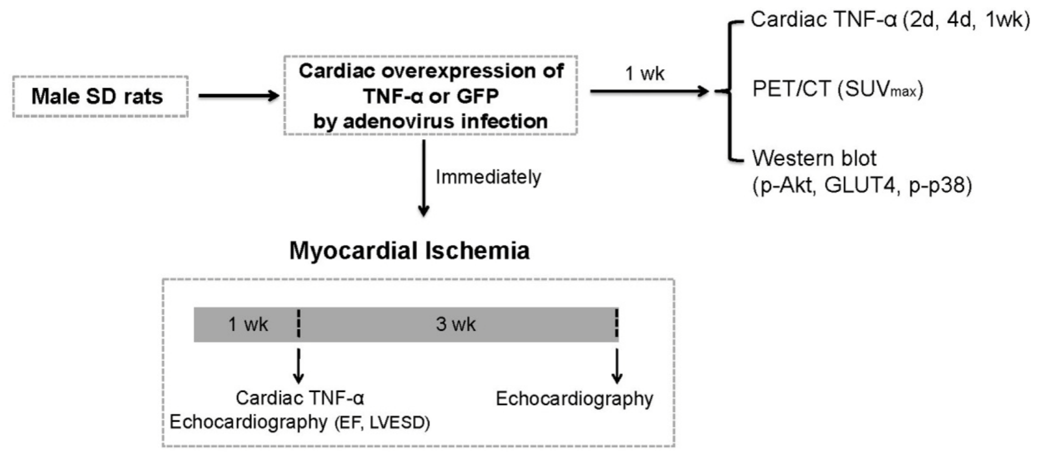

Figure 4 and 5

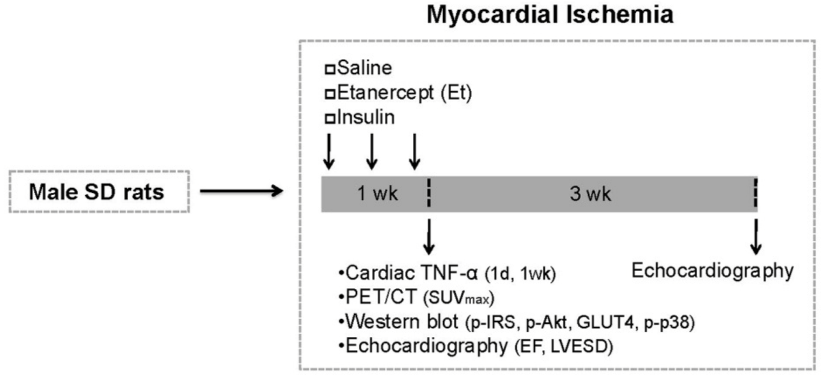

Supplement Figure S3. Schematic figure illustrating the in vivo experimental protocol of Figure 3, 4 and 5.
